# Comparison of Intravenous and Oral Meloxicam Pharmacokinetics in Female and Male Saanen Goats

**DOI:** 10.3390/vetsci12080686

**Published:** 2025-07-23

**Authors:** Zeynep Ozdemir Kutahya, Busra Aslan Akyol, Selen Mamuk, Petek Piner Benli, Cengiz Gokbulut

**Affiliations:** 1 Department of Veterinary Pharmacology and Toxicology, Faculty of Ceyhan Veterinary Medicine, Cukurova University, Adana 01330, Türkiye; zkutahya@cu.edu.tr (Z.O.K.); ppinerbenli@cu.edu.tr (P.P.B.); 2 Department of Veterinary Pharmacology and Toxicology, Institute of Health Sciences, Balikesir University, Balikesir 10145, Türkiye; busra.aslan@baun.edu.tr; 3 Department of Veterinary Pharmacology and Toxicology, Institute of Health Sciences, Cukurova University, Adana 01330, Türkiye; smamuk@student.cu.edu.tr; 4 Department of Medical Pharmacology, Faculty of Medicine, Balikesir University, Balikesir 10145, Türkiye

**Keywords:** meloxicam, goat, gender, pharmacokinetics, intravenous, oral

## Abstract

This study aimed to investigate the effect of gender on the pharmacokinetics of meloxicam in goats following intravenous (IV, 0.5 mg/kg) and oral (PO, 1.0 mg/kg) administration. A crossover design was used with 12 clinically healthy Saanen goats (six females and six males). Plasma samples were collected up to 96 h post-administration and analyzed with an HPLC for meloxicam concentrations. Pharmacokinetic parameters were calculated and statistically compared between genders and administration routes. The results show that male goats exhibited significantly longer terminal half-life (T_1/2λz_), a greater mean residence time (MRT_0–∞_), and higher systemic exposure (AUC_0–∞_) than females, particularly after oral administration. Oral bioavailability was calculated as 77.43% in females and 104.73% in males. These differences may be linked to gender-based variations in hepatic metabolism, enterohepatic recirculation, and the hormone-mediated modulation of cytochrome P450 activity. The findings are consistent with previous research demonstrating that gender can influence drug disposition through hormonal and enzymatic mechanisms. This study underscores the importance of considering gender as a biological variable in pharmacokinetic assessments of veterinary drugs, especially those used in food-producing animals, to optimize dosing strategies and ensure both therapeutic efficacy and food safety.

## Simple Summary

Meloxicam is a medication commonly used in animals to reduce pain and inflammation, helping them recover more comfortably after illness or surgery. However, the way this medication is processed in the body can differ between females and males, which may affect how long it stays in the system and how well it works. In this study, we examined how female and male goats handle meloxicam after it was given by mouth and administered directly into the bloodstream. We found that when the medicine was given by mouth, it stayed in male goats’ bodies longer and at higher levels than in females. These differences are important because they can affect how often the medication should be given and determine how long we should wait before using goat products for human consumption to ensure safety. Understanding these differences can help veterinarians make better decisions about treating pain in goats and may guide medication practices in other animals raised for food, ensuring both animal welfare and food safety.

## 1. Introduction

Non-steroidal anti-inflammatory drugs (NSAIDs), which are widely used in veterinary medicine, play a significant role, particularly in managing pain, inflammation, and fever. They are often preferred in conditions such as postoperative pain, musculoskeletal diseases (e.g., osteoarthritis and laminitis), visceral pain (e.g., colic), inflammatory diseases (e.g., pneumonia and mastitis), and fever reduction [1]. NSAIDs mainly suppress prostaglandin synthesis by inhibiting cyclooxygenase (COX) enzymes, thereby reducing pain, fever, and inflammation [2]. While COX-1 is involved in normal physiological functions, COX-2 is induced during inflammation. Consequently, COX-2 selective NSAIDs (e.g., meloxicam and firocoxib) have fewer gastrointestinal side effects [1,2]. NSAIDs may cause adverse effects, particularly in the gastrointestinal and renal systems [2]. Although NSAIDs are highly effective and essential drugs widely used in veterinary medicine, they should be administered with caution and awareness. Meloxicam belongs to the enolic acid group of NSAIDs and exhibits analgesic, anti-inflammatory, and antipyretic effects [3]. It preferentially inhibits COX-2 over COX-1, but it is not entirely COX-2 selective; thus, at higher doses, its selectivity for the COX-2 isoenzyme diminishes [4]. Meloxicam is extensively metabolized in the liver and undergoes significant enterohepatic recirculation [5]. Its metabolites have not been found to possess any pharmacological activity [6]. Meloxicam is an approved NSAID for cattle, horses, cats, and dogs, while it is used off-label in sheep and goats. Pharmacological studies increasingly highlight gender-based differences in drug absorption, distribution, metabolism, and excretion [7,8,9,10,11,12]. Incorporating gender as a crucial variable in dosage adjustment is important. Moyer et al. [13] discussed how gender chromosomes and endogenous steroid hormones modulate drug transporters, metabolic enzymes, and receptors, thereby influencing pharmacokinetics and adverse drug responses. Moreover, Bosch et al. [14] stated that female sex hormones, particularly estrogen, significantly impact drug metabolism mediated by CYP and UGT enzyme activity. These factors are especially relevant for orally administered drugs due to the influence of first-pass hepatic metabolism affected by estrogenic activity. Meloxicam is known to have high oral bioavailability (72–96%) across various species including goats, sheep, cattle, and horses, indicating minimal first-pass hepatic metabolism [15]. Data on the gender-dependent pharmacokinetics of drugs used in ruminants remain limited. It is scientifically and clinically valuable to explore how the pharmacokinetics of meloxicam, a commonly used NSAID in veterinary medicine, is affected by gender in food-producing species like goats. While the existing literature addresses gender differences in humans and laboratory animals, no prior studies have focused on meloxicam in goats. Therefore, our research aimed to assess whether and how the pharmacokinetic parameters of meloxicam differ between female and male Saanen goats following intravenous (0.5 mg/kg) and oral (1.0 mg/kg) administration.

## 2. Materials and Methods

### 2.1. Chemicals

Analytical standards of meloxicam (99.2%, CAS: 71125-38-7) and piroxicam (99.7%, CAS: 36322-90-4) were obtained from LGC Standards (Wesel, Germany). HPLC-grade acetonitrile (99.9%), methanol (99.9%), magnesium sulphate heptahydrate (99.9%), and phosphoric acid (89%) were supplied by Sigma-Aldrich (Steinheim, Germany).

### 2.2. Animals

This study involved a total of 12 Saanen goats, comprising six females and six males, all approximately one year of age. The average body weight was 28.19 ± 3.35 kg for the female goats and 36.87 ± 4.58 kg for the males. Before the experiment, each animal underwent a comprehensive clinical evaluation along with hematological testing to ensure they were in good health. None of the goats had been treated with any medication for two months prior to the experiment. The animals were divided into two groups according to gender. Throughout the study period, they were provided with commercial concentrate feed, and both dry forage and water were available *ad libitum.* The experiments with the males were performed in late autumn as the endogenous testosterone levels are at a maximum at this time. The experiments with the females were undertaken during the same period. The female goats used in this study were approximately one year old and had not yet been bred; thus, their estrous cycles were not active or regular, and monitoring cycle stages was not considered necessary. The study protocol was approved by the Ethics Committee of Cukurova University, Health Sciences Experimental Application and Research Centre.

### 2.3. Study Design

The study was conducted using a crossover design consisting of two experimental phases separated by a 10-day washout period [16,17,18] between administration routes in both female and male Saanen goats. Meloxicam was administered in two commercially available formulations: oral tablets (Melox Fort, 15 mg/tablet, Nobel Pharmaceuticals, Istanbul, Türkiye) at a dose of 1.0 mg/kg and an injectable solution (Metacam, 20 mg/mL, Boehringer Ingelheim, Istanbul, Türkiye) at a single intravenous dose of 0.5 mg/kg. The doses used in this study (0.5 mg/kg IV and 1.0 mg/kg PO) were selected based on previous pharmacokinetic studies in ruminants, aligning with commonly reported ranges while reflecting clinically relevant dosing practices [16,17,19]. In this crossover design, each animal received both the IV and PO formulations in two separate phases, with a washout period in between. All treatments were administered in the morning before feeding with a meloxicam-free commercial concentrate. Animals were closely observed for any adverse effects (behavior, emesis, salivation, diarrhea, weakness, and anorexia) following both IV and PO administration of meloxicam. Blood samples (3 mL) were collected in heparinized tubes before the treatment (0) and subsequently at 5, 15, 30, and 45 min and 1, 1.5, 2, 4, 6, 8, 10, 16, 24, 36, 48, 72, and 96 h post-administration. Blood samples were subjected to centrifugation at 4000× *g* for 7 min within 60 min of collection, and the plasma samples were stored at −80 °C until further analysis.

### 2.4. Analytical Procedure

#### 2.4.1. Instrumentation

Chromatographic analyses were performed by using an Agilent 1260 HPLC system (Agilent, Waldbronn, Germany) consisting of a binary high-pressure gradient system used for the analysis of meloxicam. An Agilent binary pump (G1312B) was used to deliver the mobile phase to the analytical column. Sample injection was performed via an Agilent autosampler (G1367E) coupled with an injection valve (Rheodyne^®^, Rohnert Park, CA, USA) equipped with a 100 µL variable loop. Detection was achieved using a diode array detector (G4212B) in compliance with the data acquisition ChemStation Software by Agilent (Germany). Degassing of the mobile phase was achieved using an Agilent vacuum degasser unit (G4225A). Operations and functions of the whole HPLC system were controlled using ChemStation^®^ Software (C.01.08, Agilent, Waldbronn, Germany).

All evaporations following sample extraction were performed at 50 °C using a vacuum evaporator (Maxi-Dry Plus, Heto Lab. Equipment, Allerod, Denmark). A vortex mixer (622, Isolab, Wertheim, Germany) and a centrifuge (Hettich Rotina^®^ 380R, Kirchlengern, Germany) were used for the extraction procedure.

#### 2.4.2. Chromatographic Conditions

The separation of meloxicam was achieved through the use of an analytical column (Eclipse XDB-C18, 5 µm, 250 mm × 4.6 mm, Agilent) with a Nucleosil C18 guard column (Phenomenex, Macclesfield, UK), which was maintained at 50 °C during analysis in a column oven (G1316A). The mobile phase was composed of ultra-pure water (H_2_O), methanol, and acetonitrile in a ratio of 40:30:30 (*v*/*v*/*v*) and adjusted to a pH of 3.5 using phosphoric acid. It was delivered isocratically at a flow rate of 1 mL/min. A photodiode array detector (G4212B) was used at a wavelength of 355 nm. The injection volume was consistently 50 µL, and each chromatographic analysis took 9 min after the injection.

#### 2.4.3. Preparation of Standard Solution

Stock analytical standard solutions (100 µg/mL) of meloxicam and the internal standard piroxicam (5 µg/mL) were prepared in a mixture of acetonitrile/ultra-pure water (50:50) and stored in glass bottles at 4 °C. The stock solution of meloxicam was diluted with a mixture of acetonitrile/ultra-pure water (50:50) to give solutions for plasma samples (0.1, 0.5, 1, 5, 10, and 50 µg/mL). These standards were used to spike drug-free samples at various concentrations to generate standard curves and determine extraction recovery rates.

#### 2.4.4. Sample Preparations and Extraction

The plasma levels of meloxicam were analyzed using HPLC with a diode array detector following liquid–liquid phase extraction procedures according to Karademir et al. [16] and Bae et al. [20] with small modifications described below. Accordingly, blank plasma samples (0.25 mL) were spiked with 25 µL of meloxicam standard solution to reach the following final concentrations: 0, 0.01, 0.05, 0.1, 0.5, 1, 5, and 10 µg/mL for the IV route and 0, 0.01, 0.05, 0.1, 0.5, and 1 µg/mL for the PO route. The Spike concentrations used in the analysis of both intravenous and oral administration samples were determined by considering the peak concentrations obtained in previous studies in the literature. The plasma samples were combined with 50 µL of internal standard (piroxicam 5 µg/mL). Subsequently, magnesium sulphate heptahydrate (0.1 g) was added, and the mixture was vortexed for 1 min. Then, 1 mL of acetonitrile was added to deproteinize the plasma samples, and the mixture was vortexed for an additional 1 min. The mixture was then centrifuged at 14,500× *g* for 5 min. The upper phase was transferred to a 10 mL tube and evaporated using a vacuum concentrator set at 50 °C. Once the residue was completely dried, it was dissolved in 200 µL of the mobile phase and vortexed for 15 s. Finally, 50 µL of this solution was injected into the chromatographic system for analysis.

#### 2.4.5. Validation of Analytical Method

Analytical validation was conducted to determine meloxicam levels in plasma samples in accordance with the International Conference on Harmonization (ICH) guidelines for validating analytical procedures [21]. The analyte was identified based on the retention times of a pure reference standard. Recoveries of the substance under study were assessed by comparing the peak areas from spiked plasma samples with those obtained from direct injections of standards prepared in a mixture of acetonitrile and ultra-pure water. The recoveries were calculated with five multiple concentrations and six replications. To evaluate the inter- and intra-assay precision of the extraction and chromatography procedures, replicate aliquots of drug-free goat plasma samples containing known amounts of meloxicam were processed on different days. A calibration graph for meloxicam was created, demonstrating a linear range of 0.01 to 10 µg/mL for IV administration and 0.01 to 1 µg/mL for PO administration. The slope of the line relating peak areas to drug concentration was determined using least squares linear regression, and both the correlation coefficient (*r*) and the coefficient of variation (CV) were calculated. Linearity was established to confirm the relationship between the meloxicam concentration and detector response. The detection limit (LOD) for meloxicam was determined through an HPLC analysis of blank plasma samples fortified with the standard. This involved measuring the baseline noise at the retention time of the peak. The LOD was defined as the mean baseline noise at the peak retention time plus three standard deviations (SDs). Additionally, the limit of quantification (LOQ) was defined as the mean baseline noise plus six SDs.

### 2.5. Pharmacokinetics and Statistical Analysis of Data

The plasma concentrations vs. time curves obtained following each administration route in individual animals were fitted with the WinNonlin software program (Version 5.2, Pharsight Corporation, Mountain View, CA, USA). The pharmacokinetic parameters for each animal were analyzed using non-compartmental model analysis. The maximum plasma concentration (C_max_) and time to reach maximum concentration (T_max_) were obtained from the plotted concentration–time curve of the drug in each animal. The trapezoidal rule was used to calculate the area under the plasma concentration–time curve (AUC). The fraction of dose absorbed (i.e., *F*) was calculated with the use of mean AUCs calculated for each route of administration using the following equation after dose normalization:

*F* = (AUC_PO_/AUC_IV_) × (0.5/1) × 100

Pharmacokinetic parameters are presented as mean ± SD. Harmonic means were calculated for T_1/2λz_, MRT_last_, and MRT_0–∞_. For the comparison of administration routes, non-normally distributed data were analyzed using the non-parametric Wilcoxon signed-rank test, while normally distributed data were assessed using the paired *t*-test. When comparing gender, non-normally distributed data were compared with the non-parametric Mann–Whitney U test, and normally distributed data were analyzed using the independent *t*-test. All statistical analyses were performed using SPSS Statistics version 23.0 (IBM Corp).

## 3. Results

The validation parameters of meloxicam for the analysis of plasma samples of goats are given in Table 1.

Animals were closely observed for adverse effects, including behavior, emesis, salivation, diarrhea, weakness, and anorexia, throughout the 20-day study period following both IV and PO administration of meloxicam. Gender-related differences were observed in several pharmacokinetic parameters following IV and PO administration of meloxicam at doses of 0.5 mg/kg and 1.0 mg/kg, respectively, in Saanen goats (Table 2). Although the initial plasma concentration (C_0_), area under the curve (AUC_0–∞_), clearance (Cl), mean residence time (MRT), and area under the first moment curve (AUMC) values were similar between female and male goats, the terminal elimination half-life (T_1/2λz_) was notably longer in males (10.09 ± 0.97 h vs. 11.72 ± 0.74 h) after IV administration. Following PO administration of meloxicam at a dose of 1.0 mg/kg, notable pharmacokinetic differences were observed between female and male Saanen goats. Although the maximum plasma concentration (C_max_) and time to reach C_max_ (T_max_) were similar across genders, significant differences emerged in elimination and exposure parameters. The T_1/2λz_ was significantly longer in male goats (13.10 ± 2.01 h) compared to females (9.87 ± 0.85 h). In parallel, MRT_0–∞_ was also significantly prolonged in males (22.18 ± 3.47 h) relative to females (17.12 ± 1.73 h). Significant differences were observed between the pharmacokinetic parameters obtained after intravenous (IV, 0.5 mg/kg) and oral (PO, 1.0 mg/kg) administration of meloxicam to female and male goats. No difference in mean T_1/2λz_ values was observed between IV and PO meloxicam administration in both female and male goats. The AUC, AUMC, and MRT parameters were determined to be significantly higher after PO administration. It was also determined that meloxicam has a high bioavailability (*F*) when administered orally in female (77.43%) and especially male goats (104.73%).

**Figure 1:**
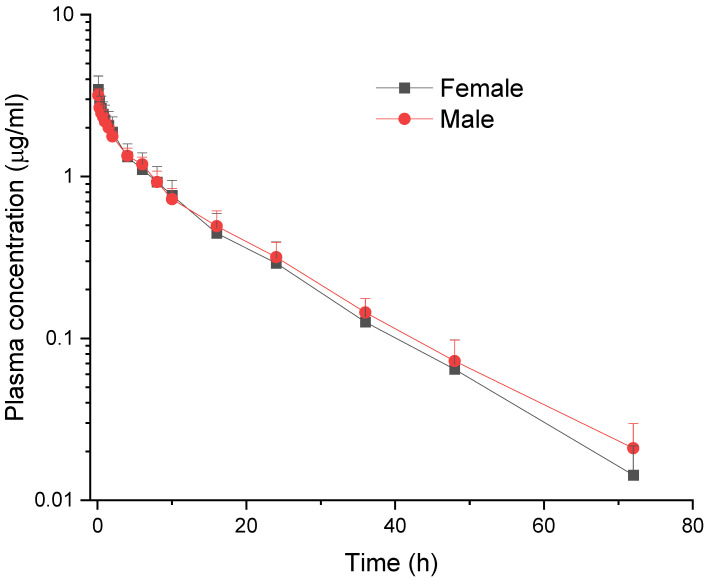
Comparative mean (±SD) plasma concentration vs. time curves of meloxicam in female and male goats following intravenous administration at a dose of 0.5 mg/kg (*n* = 6).

**Figure 2:**
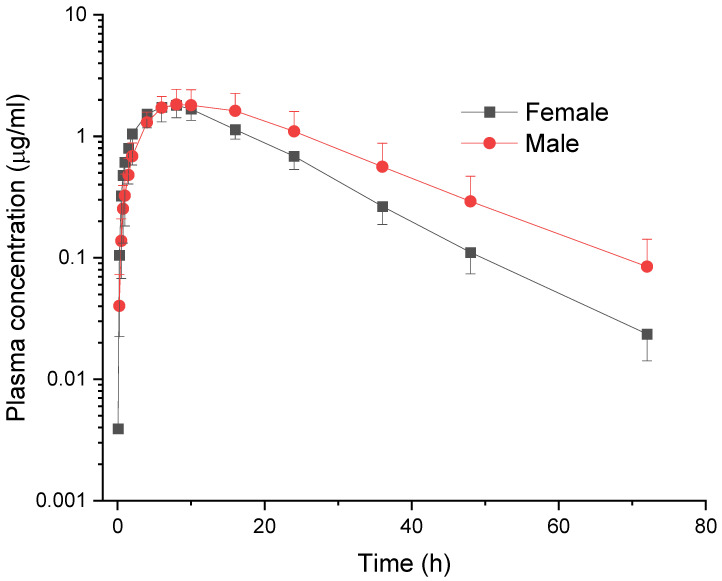
Comparative mean (±SD) plasma concentration vs. time curves of meloxicam in female and male goats following per os administration at a dose of 1.0 mg/kg (*n* = 6).

Meloxicam was detected in plasma for approximately 70 h in female and male goats after IV (Figure 1) and PO administration (Figure 2).

## 4. Discussion

It is imperative to comprehend how gender influences drug disposition to optimize therapeutic strategies, ensure animal welfare, and refine dosage recommendations, particularly in food-producing species such as goats. In both genders, pharmacokinetic variability can have both clinical and regulatory implications. The present study was conducted to evaluate the pharmacokinetics of meloxicam, a commonly used NSAID in veterinary medicine, in male and female Saanen goats following both IV and PO administration.

Following IV administration of meloxicam at a dose of 0.5 mg/kg, a statistically significant difference was observed in the T_1/2λz_ values between female and male goats. The T_1/2λz_ was notably longer in males (11.72 ± 0.74 h) than in females (10.09 ± 0.97 h), suggesting that meloxicam is cleared more slowly in male goats. This prolonged elimination could reflect gender-based differences in hepatic metabolism, protein binding, or renal excretion rates. Although the estrous cycle was not active in the female goats used in this study due to their age and breeding status, gender-based differences in pharmacokinetics can still occur. These differences may result from baseline hormonal variations, including higher testosterone levels in males during late autumn, and from physiological factors such as plasma protein binding, body composition, and renal clearance, all of which can influence drug disposition independently of the estrous cycle. Despite the absence of statistically significant differences in other pharmacokinetic parameters, such as C_0_, AUC_0–∞_, Cl, and volume of distribution (V_ss_), between the genders, the consistently higher MRT_0–∞_ and AUMC values observed in males provide further evidence that supports the hypothesis of slower drug disposition in males. These findings indicate that, despite similar initial plasma concentrations and exposure levels, meloxicam remains in the system for longer in male goats. It is imperative to consider the implications of this prolonged systemic persistence on both therapeutic efficacy and withdrawal time considerations. A recent study investigated the pharmacokinetic differences in meloxicam between male and female sheep following IV administration. The results show that the total clearance (Cl_T_) and Vdss were significantly higher in male sheep, while the T_1/2λz_ was significantly shorter compared to female sheep [11]. In our study, meloxicam was administered at a dose of 0.5 mg/kg IV to Saanen goats, whereas Corum et al. [11] employed a 1.0 mg/kg IV dose in Romanov sheep. Despite this difference in dosing, both studies reveal that gender significantly influences meloxicam disposition, particularly in terms of T_1/2λz_. In male goats, the T_1/2λz_ (11.72 ± 0.74 h) was longer than that observed in male sheep (9.47 ± 0.25 h), while female goats had a slightly shorter T_1/2λz_ (10.09 ± 0.97 h) than female sheep (11.96 ± 0.33 h). The fact that the T_1/2λz_ is longer in male goats than in male sheep is a surprising result because studies have shown that goats metabolize and eliminate compounds faster than sheep [22,23,24,25]. Although male sheep exhibited faster Cl (8.72 ± 1.34 mL/h/kg) and larger Vd (100.95 ± 14.73 mL/kg) than females, such pronounced differences were not observed in the goats. In our study, both Cl and Vd values were similar across genders, suggesting that species-related physiological or metabolic factors may play a more prominent role in drug disposition than gender alone in goats. Another notable distinction concerns the extent of systemic exposure. In sheep, female animals showed significantly higher AUC_0–∞_ values (224.68 ± 28.22 μg·h/mL) compared to males (117.25 ± 20.38 μg·h/mL), indicating a marked gender-based difference in meloxicam exposure. In contrast, in goats, AUC values were comparable between genders (females: 25.57 ± 6.76 vs. males: 26.43 ± 4.30 μg·h/mL), further underscoring the interspecies variability in meloxicam pharmacokinetics. These differences could be attributed not only to species-specific hepatic enzyme activity (such as CYP2C9-mediated metabolism) but also to variations in plasma protein binding, tissue distribution, and renal excretion patterns between goats and sheep. Nevertheless, direct comparisons should be interpreted with caution due to differences in administered doses (0.5 mg/kg in goats vs. 1.0 mg/kg in sheep), species-related physiological variations (Saanen goats vs. Romanov sheep), and potential differences in study design, all of which may influence pharmacokinetic parameters. Both studies highlight the importance of gender in determining the pharmacokinetics of meloxicam; however, the results demonstrate that these effects are species dependent.

In the present study, gender-related pharmacokinetic differences were also observed in Saanen goats following a single PO administration of meloxicam at a dose of 1.0 mg/kg. Male goats exhibited a significantly longer T_1/2λz_ (13.10 ± 2.01 h) compared to females (9.87 ± 0.85 h), indicating slower elimination. This was further supported by the longer MRT_0–∞_ observed in males (22.18 ± 3.47 h vs. 17.12 ± 1.73 h in females), suggesting an extended systemic persistence of meloxicam in male animals. In addition, although C_max_ was similar between genders, the total drug exposure represented by AUC_0–∞_ was higher in males (55.36 ± 22.38 μg·h/mL) than in females (39.59 ± 7.45 μg·h/mL), although not statistically significant, but with a high degree of inter-individual variability. These findings suggest that meloxicam is more systemically available in males than females following PO administration in goats. Meloxicam is subject to complete metabolic conversion to four pharmacologically inactive metabolites, primarily through the cytochrome P450 2C pathway [26]. Thus, the observed differences in elimination and exposure in male and female animals may be attributable to gender-based variations in hepatic enzyme activity, enterohepatic recirculation, gastrointestinal transit times, or hormonal influences affecting drug metabolism and excretion.

In this study, the IV and PO routes were selected to evaluate the pharmacokinetics and calculate the absolute *F* of meloxicam in goats, as the IV route serves as the reference for *F* calculations. Although the IM route is commonly used in veterinary practice, it was not included in this study to maintain methodological clarity for *F* assessment and to focus on the establishment of pharmacokinetic parameters relevant to dosage regimen design in food-producing animals. A thorough evaluation of meloxicam pharmacokinetics after IV and PO administration in both female and male Saanen goats shows significant differences in drug disposition based on the route of administration and gender. While the T_1/2λz_ values remained generally consistent across genders and administration routes—suggesting similar elimination kinetics once meloxicam enters the systemic circulation—statistically significant differences emerged in drug exposure and MRT, especially following PO administration. In both genders, PO administration of meloxicam at 1.0 mg/kg resulted in significantly higher systemic exposure compared to 0.5 mg/kg IV administration. This was evident in the AUC_0–∞_ values, which increased from 25.57 ± 6.76 to 39.59 ± 7.45 μg·h/mL in females and from 26.43 ± 4.30 to 55.36 ± 22.38 μg·h/mL in males. Correspondingly, AUMC_0–∞_ and MRT_0–∞_ values were significantly elevated after PO dosing in both genders, indicating a prolonged systemic presence of meloxicam via the oral route. Notably, the increase in MRT_0–∞_ from IV to PO was more pronounced in males (14.27 h vs. 22.18 h) than in females (12.77 h vs. 17.12 h), suggesting that male goats may exhibit a slower drug turnover and more sustained drug retention after PO administration. Interestingly, the calculated absolute *F* of oral meloxicam was 77.43% in females and 104.73% in males. While the high *F* value in males may partly reflect enterohepatic recirculation, it may also be influenced by the sex hormone-mediated modulation of hepatic enzyme activity, differences in gastrointestinal physiology affecting absorption efficiency, and potential variations in plasma protein binding between genders in addition to inter-individual variability. Although dose normalization was applied in *F* calculation, potential nonlinear pharmacokinetics at the administered dose cannot be completely excluded as a contributing factor. The consistent T_max_ values (7.33 ± 1.03 h in females vs. 8.33 ± 1.51 h in males) suggest a delayed yet efficient absorption profile for oral meloxicam across both genders, with slightly slower absorption kinetics in males. From a clinical standpoint, these findings underscore that although both genders achieve therapeutic plasma levels of meloxicam after PO administration, male goats may maintain these levels for a longer duration due to prolonged MRT and higher AUC. While T_1/2λz_ remained unaffected by the administration route, the overall pharmacokinetic behavior was modulated by both the administration route and gender, reinforcing the need to account for these variables in designing dosage regimens, determining dosing intervals, and establishing withdrawal periods for food-producing animals. The therapeutic plasma concentration of meloxicam in goats has not been definitively established in this study, although data from related ruminant species suggest that maintaining plasma levels above a certain threshold is essential for effective anti-inflammatory and analgesic effects [27,28,29]. The prolonged MRT and higher systemic exposure observed in male goats suggest that dosing intervals might be extended compared to females to maintain therapeutic levels while minimizing drug accumulation and potential toxicity.

The gender-related pharmacokinetic differences observed in meloxicam disposition in goats may be partially attributed to hormonal modulation of drug metabolism. Previous studies in goats have shown that females can eliminate compounds like antipyrine more rapidly, while males may exhibit slower elimination, suggesting a gender influence on hepatic enzyme activity [30]. Additionally, testosterone administration in dwarf goats has been shown to suppress the formation of specific oxidative antipyrine metabolites, indicating hormonal modulation of hepatic metabolism [31]. Similarly, studies with ivermectin reported longer T_1/2_ and MRT in male goats despite similar C_max_ and AUC values, indicating slower drug elimination in males [12]. Hormonal influences, particularly testosterone in males, may suppress hepatic oxidative metabolism and contribute to prolonged drug exposure, while estrogen in females may enhance CYP3A activity and drug clearance [31,32]. Differences in gastrointestinal physiology such as motility and pH, as well as body composition and renal function differences between genders, may further explain the observed differences in *F* and MRT [13,14,33,34]. These physiological and hormonal factors can impact the pharmacokinetics of orally administered drugs, aligning with our findings of higher bioavailability, prolonged MRT, and elevated AUC in male goats, especially after PO meloxicam administration. Our results, together with previous studies highlighting the clinical impact of gender-related pharmacokinetic differences, support the importance of considering gender when designing dosing regimens and withdrawal times for meloxicam in goats to ensure optimal efficacy and food safety [35,36,37].

While this study provides valuable insights into the gender-related pharmacokinetics of meloxicam in Saanen goats, certain limitations should be noted. The investigation was limited to single-dose administration under controlled conditions in a single breed, which may affect extrapolation to other breeds or field settings. Meloxicam metabolites and hepatic enzyme activities were not assessed, which could clarify the mechanisms underlying the observed gender differences. Additionally, potential influences of body weight-related physiological differences were not separately analyzed. Future studies incorporating repeated dosing, pharmacodynamic evaluation, and residue depletion data will be essential to translate these pharmacokinetic findings into clinical and regulatory practice.

Taken together, these findings underscore the need to incorporate gender as a biological variable in pharmacokinetic studies, particularly for veterinary drugs administered orally in food-producing animals. In particular, the longer MRT and higher systemic exposure observed in male goats indicate that male animals may require extended withdrawal periods or adjusted dosing intervals to avoid violative residues while maintaining therapeutic efficacy. The integration of the findings in the literature with our data suggests that sex hormones can affect the activity of hepatic enzymes involved in meloxicam metabolism, which may lead to clinically relevant differences in drug exposure and clearance. These findings could have significant implications for adjusting dosage regimens and withdrawal periods to ensure both therapeutic efficacy and food safety.

## Figures and Tables

**Table 1:** Validation parameters of the analytical method used to determine meloxicam in plasma samples.

Parameters	Meloxicam
LOD (µg/mL)	0.0031
LOQ (µg/mL)	0.01
Range of linearity (µg/mL)	0.01–10.00
Linearity (*r*^2^)	0.9987–1.0000
Recovery (%)	85.85 (2.91)
Coefficient of variation (%)	4.51 (0.73)

LOD: limit of detection, LOQ: limit of quantification, *r*: correlation coefficient. The values in the brackets represent the standard deviations for the recovery assays (*n* = 6).

**Table 2:** Pharmacokinetic parameters of meloxicam after intravenous (IV, 0.5 mg/kg) and oral (PO, 1.0 mg/kg) administration to female and male goats (*n* = 6).

Parameters	IV Administration		PO Administration	
	Female	Male	Female	Male
T_1/2λz_ (h) ^a^	10.09 ± 0.97	11.72 ± 0.74 *	9.87 ± 0.85	13.10 ± 2.01 *
C_0_ (µg/mL)	3.76 ± 0.82	3.47 ± 0.39	-	-
T_max_ (h)	-	-	7.33 ± 1.03	8.33 ± 1.51
C_max_ (µg/mL)	-	-	1.87 ± 0.38	1.90 ± 0.50
AUC_last_ (µg.h/mL)	25.35 ± 6.65	26.07 ± 4.14	39.24 ± 7.36	53.60 ± 21.12
AUC_0–∞_ (µg.h/mL)	25.57 ± 6.76	26.43 ± 4.30	39.59 ± 7.45	55.36 ± 22.38
Vz (mL/kg)	302.08 ± 66.15	325.47 ± 36.97	-	-
Cl (mL/h/kg)	20.69 ± 5.20	19.30 ± 2.83	-	-
AUMC_last_ (µg.h^2^/mL)	319.66 ± 113.72	351.23 ± 82.09	654.61 ± 141.85	1147.71 ± 556.79
AUMC_0–∞_ (µg.h^2^/mL)	338.73 ± 124.71	383.57 ± 97.28	684.96 ± 153.68	1311.62 ± 685.47
MRT_last_ (h) ^a^	12.22 ± 1.50	13.30 ± 0.97	16.55 ± 1.52	20.35 ± 2.55 *
MRT_0–∞_ (h) ^a^	12.77 ± 1.74	14.27 ± 1.26	17.12 ± 1.73	22.18 ± 3.47 *
V_ss_ (L/kg)	263.27 ± 52.26	274.50 ± 24.70	-	-
*F* (%)	-	-	77.43	104.73

T_1/2λz_: terminal half-life; C_0_: calculated concentration at time zero of IV administration; T_max_: time to reach peak plasma concentration; C_max_: peak plasma concentration; AUC: area under (zero moment) curve from time 0 to last detectable concentration; Vz: volume of distribution; Cl: clearance of drug; AUMC: area under moment curve from time 0 to t last detectable concentration; MRT: mean residence time; V_ss_: apparent volume of distribution at steady state; *F*: bioavailability. ^a^ Harmonic mean. * Kinetic parameters in male goats are significantly different (*p* < 0.05) from those in female goats.

## Data Availability

The data presented in this study are available within the article’s figures and tables.
